# Effects of Non-invasive Neuromodulation on Executive and Other Cognitive Functions in Addictive Disorders: A Systematic Review

**DOI:** 10.3389/fnins.2018.00642

**Published:** 2018-09-19

**Authors:** Renée S. Schluter, Joost G. Daams, Ruth J. van Holst, Anna E. Goudriaan

**Affiliations:** ^1^Department of Psychiatry and Amsterdam Institute for Addiction Research, Amsterdam UMC, University of Amsterdam, Amsterdam, Netherlands; ^2^Medical Library, Amsterdam UMC, University of Amsterdam, Amsterdam, Netherlands; ^3^Donders Institute for Cognition, Brain and Behaviour, Radboud University, Nijmegen, Netherlands; ^4^Arkin, Department of Care, Research and Quality of Care, Amsterdam, Netherlands

**Keywords:** non-invasive neuromodulation, transcranial direct current stimulation, repetitive transcranial magnetic stimulation, prefrontal cortex, executive functioning, cognitive functioning, substance use disorders

## Abstract

**Background:** In order to improve the current treatment of addictive disorders non-invasive neuromodulation over the dorsolateral prefrontal cortex (DLPFC) has gained attention. The DLPFC is crucially involved in executive functioning, functions which are related to the course of addictive disorders. Non-invasive stimulation of the DLPFC may lead to changes in executive functioning. Currently an overview of effects of neuromodulation on these functions is lacking. Therefore, this systematic review addresses the effects of non-invasive neuromodulation on executive functioning in addictive disorders.

**Methods:** The current review is conducted and reported in accordance with the Preferred Reporting Items for Systematic reviews and Meta-Analyses for Protocols 2015 (PRISMA-P 2015) guidelines and has been registered in PROSPERO International Prospective Register of Systematic Reviews (https://www.crd.york.ac.uk/prospero/, registration number: CRD42018084157). Original articles were searched using the Ovid MEDLINE, Embase and PsycINFO database.

**Results:** The systematic search resulted in 1,228 unique studies, of which sixteen were included in the current review. Some of these studies do not address the classic definition of executive functions, but another cognitive function. However, they were included in this review since the field is small and still under development and we aim to give an inclusive overview in its broadest sense. The following executive and other cognitive functioning domains were assessed: attention, cognitive flexibility, response inhibition, memory and learning, problem solving, social cognition, risk taking, cognitive bias modification and overall executive functioning. The executive function domain most positively affected was social cognition followed by memory & learning, response inhibition, cognitive flexibility and attention.

**Conclusions:** The studies addressed in the current review used a large variability of stimulation protocols and study designs which complicates comparability of the results. Nevertheless, the results of these studies are promising in light of improvement of current treatment. Therefore, we recommend future studies that compare the effect of different types of stimulation, stimulation sides and number of stimulation sessions in larger clinical trials. This will significantly increase the comparability of the studies and thereby accelerate and clarify the conclusion on whether non-invasive neuromodulation is an effective add-on treatment for substance dependence.

## Introduction

### Rationale

Worldwide over 15 million people are suffering from an addictive disorder (WHO, 2010). Addictive disorders are characterized by continued substance use or gambling despite the awareness of its negative consequences (American Psychiatric Association, 2013). Currently treatment of addictive disorders consists of psychosocial interventions alone, or combined with pharmacological interventions (McHugh et al., 2010). However, similar to effectiveness of treatments for other psychiatric disorders, these treatments are only moderately effective with relapse rates of 50% within 1 year after treatment (Brandon et al., 2007; Oudejans et al., 2012). In an attempt to improve the current treatment of addictive disorders non-invasive neuromodulation has gained attention as a novel add-on treatment (Jansen et al., 2013; Bellamoli et al., 2014).

From a neurobiological perspective, addictive disorders are characterized by increased activity of the subcortical reward system in response to drug related cues, and decreased activity and functioning of the prefrontal cognitive control network (Koob and Volkow, 2013). At a behavioral level this is represented by an intense urge for the addictive substance, also referred to as craving (Sinha, 2013), and diminished cognitive control, respectively (Koob and Volkow, 2013). The imbalance between increased bottom-up (subcortical) urges combined with weakened top-down (prefrontal) neural processes often lead to relapse (Everitt and Robbins, 2005; Baler and Volkow, 2006; Wilcox et al., 2014). Non-invasive neuromodulation techniques may be able to interfere and rebalance these disturbed neurobiological processes, ultimately reducing relapse.

Two well-known non-invasive neuromodulation techniques are transcranial direct current stimulation (tDCS) and transcranial magnetic stimulation (TMS) (Hone-Blanchet et al., 2015). With tDCS two surface sponge electrodes are attached to the scalp with a rubber band. After electrode placement a low amplitude direct current runs in between the electrodes, passing through the scalp and intermediate neural tissue and thereby altering the resting membrane neuronal potential and consequently the level of excitability (Wagner et al., 2007). With anodal stimulation neurons are depolarized, leading to increased excitability (positive stimulation), whereas with cathodal stimulation neurons are hyperpolarized, leading to decreased excitability (negative stimulation) (Nitsche and Paulus, 2000). With TMS an alternating current, running through an electromagnetic coil, generates a magnetic field. When the coil is placed over the scalp the magnetic field passes the scalp and induces current in the perpendicular brain tissues. With repetitive TMS (rTMS), multiple pulses are offered in trains (Guse et al., 2010; Bellamoli et al., 2014). High frequency (HF) stimulation results in an excitatory effect on the brain tissue while low frequency (LF) works inhibitory. A subtype of high frequent stimulation is theta burst stimulation (TBS), which applies high frequent (50 Hz) pulses. With intermittent TBS (iTBS) the effect is excitatory while with continuous TBS (cTBS) the effect is inhibitory (Oberman et al., 2011). For tDCS as well as rTMS the dorsolateral prefrontal cortex (DLPFC), an important area within the cognitive control network (Ridderinkhof et al., 2004) is a frequently chosen target area.

Despite the methodological differences between rTMS and tDCS, both stimulation techniques are being investigated as add-on treatment for addictive disorders. As previously described the prefrontal cortex exerts top down control over the reward areas via corticostriatal loops (Morein-zamir and Robbins, 2014). In line with this neurobiology, an extensive body of literature indicates that rTMS and tDCS decrease craving levels after one session of tDCS or rTMS over the prefrontal cortex (for reviews see: Herremans and Baeken, 2012; Jansen et al., 2013; Bellamoli et al., 2014; Gorelick et al., 2014; Hone-Blanchet et al., 2015; Maiti et al., 2017; Trojak et al., 2017). Moreover, the prefrontal cortex is crucially involved in executive functioning (Cole and Schneider, 2007). Executive functions refer to a set of processes that guide thoughts and behaviors toward achievement of a goal (Miller, 2000) and are frequently used in order to manage situations wherein routine behavior is not sufficient to perform optimally or wherein top down control is required to correctly modify behavior (Leh et al., 2010). This set of cognitive domains includes, but is not limited to: sustained attention, initiation of goal-directed behavior, inhibition of inappropriate responses or actions (also referred to as response inhibition), flexibility to switch between rules (also referred to as cognitive flexibility), working memory, selecting relevant sensory information and abstract thinking (Leh et al., 2010; Niendam et al., 2012). Above all, these skills are required in order to succeed in substance dependence treatment, as cognitive behavioral treatment for instance requires the goal-directed selection and execution of strategic behavior required to deal with control over urges related to substance use (Blume and Alan Marlatt, 2009). However, executive functions are found to be impaired in addictive disorders (Verdejo-García et al., 2006; Wilcox et al., 2014), and have been related to a higher chance for relapse (for review see Verdejo-García et al., 2006).

After non-invasive stimulation of the prefrontal cortex one may expect changes in executive functioning depending on whether the stimulation was excitatory or inhibitory. So far, in addictive disorders the effects of neuromodulation on executive functions have only been studied scarcely, which is surprising since executive functions are impaired in substance use disordered groups and since diminished executive functions have been associated with higher relapse rates (Verdejo-García et al., 2006; Wilcox et al., 2014). Treatment outcomes (i.e., relapse rates) could therefore potentially be improved by enhancing executive functioning (Trojak et al., 2017) through non-invasive neuromodulation techniques. There are some studies in heavy substance users or individuals suffering from an addictive disorder, assessing the effects of non-invasive neuromodulation over the prefrontal cortex on one or multiple aspects of executive functioning. However, an overview of the results of these studies is currently lacking.

### Objective

To discuss the current evidence of the effect of non-invasive neuromodulation on executive functioning in addictive disorders.

### Research question

What is the effect of non-invasive neuromodulation on executive functions in individuals with an addictive disorder?

## Methods

### Study design

The current review is conducted and reported in accordance with the Preferred Reporting Items for Systematic reviews and Meta-Analyses for Protocols 2015 (PRISMA-P 2015) guidelines (Moher et al., 2015) and has been registered in PROSPERO International Prospective Register of Systematic Reviews (https://www.crd.york.ac.uk/prospero/, registration number: CRD42018084157). The PRISMA-P 2015 checklist is provided in online Supplementary Data Sheet 1.

### Participants, interventions, comparators

Participants included in the current review were persons aged between 18 and 70 who were substance users, heavy/excessive substance users or persons with a current diagnosis of a substance use disorder or gambling disorder. The intervention studied consisted of at least one session of tDCS or rTMS over an area located in the prefrontal cortex. Sham-controlled studies are included, but also studies comparing the intervention with treatment as usual, or comparing performance before and after the intervention were included. Furthermore, at least one behavioral task measured at baseline and after the neuromodulation assessing at least one aspect of executive or other cognitive functions should have been conducted, including a task performance measure. Or in case no separate behavioral tasks were reported in a study, but a combined measure of executive or other cognitive functions was presented, these studies were also included. Poster or presentation abstracts and case studies were excluded from this review.

### Systematic review protocol

Titles and abstract of studies resulting from the search were independently screened for eligibility by authors RS and RvH. Any discrepancies in judgement for eligibility were discussed until agreement by the reviewers was reached. Subsequently all selected papers were read in full to check for all inclusion criteria. In case the search revealed duplicate publications only one was included in the review.

### Search strategy

Before starting the current review we searched for ongoing studies with the same scope in the WHO international clinical trial registry platform (WHO ICTRP) and the Clinical.gov databases, but found no ongoing studies. Original articles were searched using the Ovid MEDLINE, Embase and PsycINFO databases. The search was conducted in October 2017. A scoping search identified the following key concept [] combinations which can be summarized as follows: ([prefrontal cortex] AND [non-invasive neuromodulation] AND [substance abuse] OR [prefrontal cortex] AND [non-invasive neuromodulation] AND [executive functioning]). These key concepts were translated into searches adapting (controlled) terms, database specific search fields and syntaxes belonging to the different bibliographic databases. The search was not limited to a specific time in history. See online Supplementary Data Sheet 2 for the detailed search strategy.

### Data sources, studies sections, and data extraction

The following data were extracted from the included papers by RS and MZ independently: substance and clinical status (in treatment or not), number of participants in the substance disorder group and distribution of males and females, mean age and standard deviation, the task used to assess executive functioning, outcomes defining executive functioning, the specific neuromodulation technique used and stimulation site, stimulation parameters, number of stimulation sessions and the results of any active vs. sham comparison.

Three raters (JD, HT, FW) independently assessed methodological rigor, selection and reporting bias of all included studies. For the current review the PEDro checklist (Maher et al., 2003) was used. This checklist contains 11 items which can all be scored “1” for yes and “0” for no. In case of discrepancy between the individual raters the final score was assigned based on the majority of scores (i.e., two out of three zero scores results in a final score of 0). Raters agreed on 77% of all items. The quality of the paper was considered “high” in case of 8–11 points, “medium” in case of 4–7 points and “low” in case of 1–3 points.

## Results

### Study selection and characteristics

**Figure 1:**
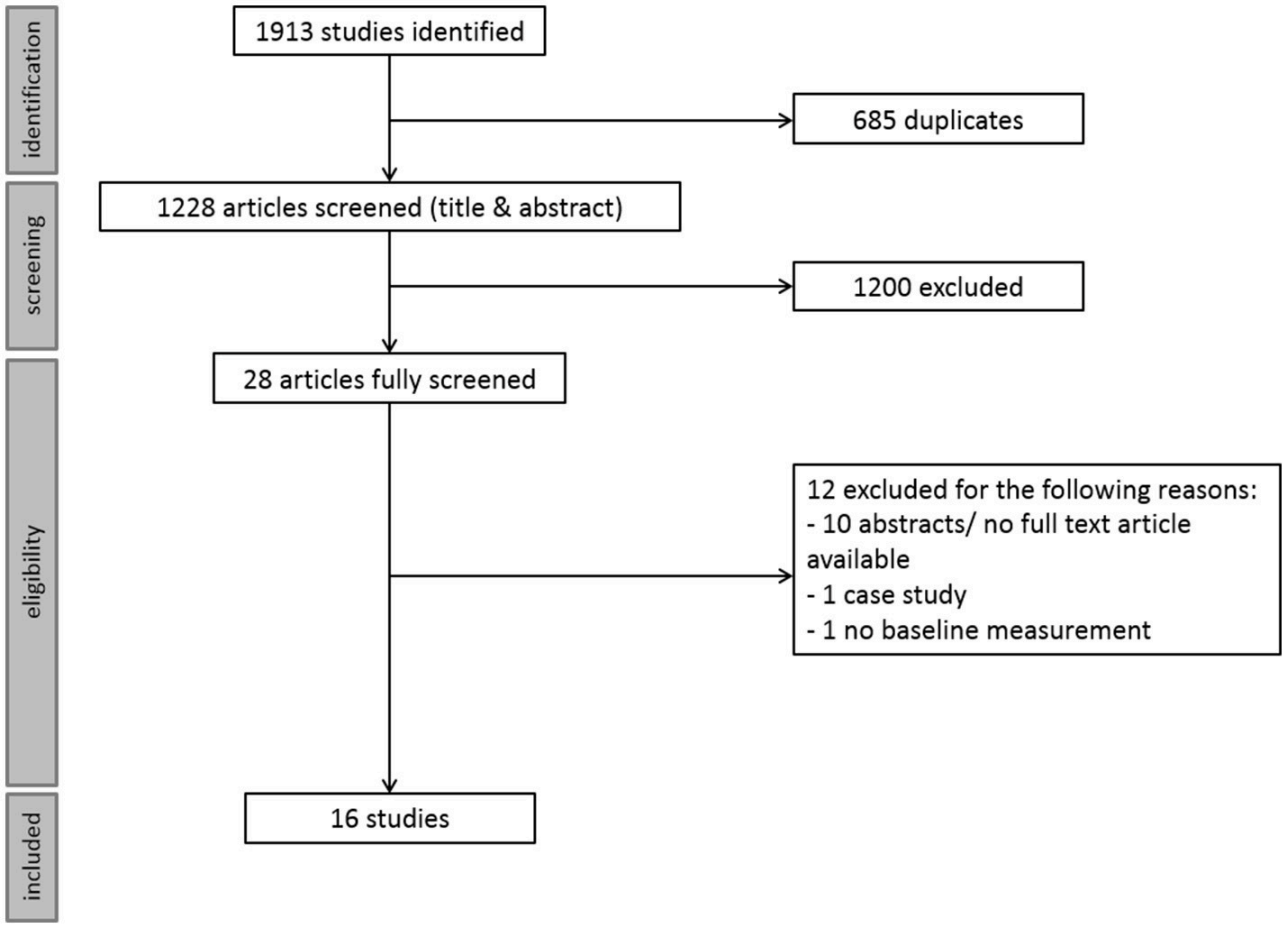
Schematic overview of the screening and inclusion procedure.

The systematic search revealed 1,228 unique studies. Screening for eligibility criteria resulted in 16 included studies. The main reason for exclusion was a study population of healthy individuals instead of a substance using or substance use disordered population. For a schematic overview of the selection process see Figure 1. Rating for methodological rigor, selection and reporting bias resulted in 9 high and 5 medium quality papers. See online Supplementary Table 1 for the score per question, and Table 1 for the final scores. All included studies were placebo controlled, and assessed executive functioning directly after the neuromodulation intervention. If the search also resulted in studies that did not fit the classic definition of executive functioning (as described in the introduction), but another cognitive function, the study was nevertheless included since the field is small and we wanted to provide a comprehensive overview of the literature. Hence, we provide an inclusive overview of the effects of non-invasive neuromodulation on executive and other cognitive functions.

**Table 1:** Studies resulting from the systematic search organized by executive functioning domain.

**Executive functioning domain**	**Study**	**Substance**	***N* (♂-♀)**	**Age [mean (*SD*)]**	**Task**	**Outcome**	**Technique**	**Stimulation parameters**	**No. of sessions**	**Results**	**PEDro scores**
Attention	Xu et al., 2013	Nicotine	24 (21–3)	45 (7.6)	Visual attention	Reaction time	tDCS (left)	Anodal lDLPFC, reference right supra-orbital area, 35 cm^2^, 2 mA, anodal 0.057 mA/cm^2^, 20 min	1	No effect	Medium
						Hit rate				No effect	
	Herremans et al., 2013	Alcohol (clinical)	29 (19–10)	48.15 (9.32)	Go-Nogo task	Commisssion errors	HF-rTMS (right)	20 Hz, 110% RMT, 40 trains of 1.9 s	1	No effect	Medium
						Mean reaction time Go trials				No effect	
						Intra-individual reaction time variability				Positive effect	
Cognitive flexibility	Huang et al., 2016	Nicotine comorbid schizophrenia sample (no intention to reduce smoking)	37 (37–0)	40.58 (3.01)^#^	Wisconsin card sorting test	Total errors	HF-rTMS (left)	10 Hz, 110% MT, 20 trains of 10 s	21	No effect	High
	Del Felice et al., 2016	Alcohol (clinical)	17 (13–4)	44.7 (unknown)	Stroop task	Accuracy in incongruent condition	HF-rTMS (left)	10 Hz, 100% RMT, 20 trains of 5 s	4	Positive effect	High
Response inhibition	Del Felice et al., 2016	Alcohol (clinical)	17 (13–4)	44.7 (unknown)	Go-NoGo task	Mean accuracy	HF-rTMS (left)	10 Hz, 100% RMT, 20 trains of 5 s	4	Positive effect	High
	Sheffer et al., 2013	Nicotine	16 (unknown)	Unknown	Delay discounting task monetary gains	Discounting rate	HF-rTMS (left)	10 Hz & 20 Hz, 110% RMT, 90 trains & 45 trains ^∧∧^	1	Positive effect	Medium
					Delay discounting task cigarette gains					Positive effect	
					Delay discounting task monetary losses					Negative effect	
					Delay discounting task cigarette losses					No effect	
Memory and learning	Su et al., 2017	Methamphetamine (clinical)	30 (30–0)	32.35 (4.96)	International shopping list task	Total number of correct responses	HF-rTMS (left)	10 Hz, 80% RMT, 24 trains of 5 s	5	Positive effect	High
					N-back Task	Proportion of correct responses				No effect	
					Continuous paired association learning task	Total number of errors				No effect	
	Qiao et al., 2016	Alcohol (clinical)	38 (25–13)	Active: 49^##^Sham: 48^##^	Hopkins verbal learning test revised	Total score	HF-rTMS (right)	10 Hz, 80% RMT, 8 trains of 10 s	4	Positive effect	High
					Brief visuospatialmemory test revised					Positive effect	
Problem solving	Su et al., 2017	Methamphetamine (clinical)	30 (30–0)	32.35 (4.96)	Groton maze learning task	Total number of errors	HF-rTMS (left)	10 Hz, 80% RMT, 24 trains of 5 s	5	No effect	High
Social cognition	Su et al., 2017	Methamphetamine (clinical)	30 (30–0)	32.35 (4.96)	Social emotionalcognition task	Proportion of correct responses	HF-rTMS (left)	10 Hz, 80% RMT, 24 trains of 5 s	5	Positive effect	High
Risk taking	Pripfl et al., 2013	Nicotine	18 (8–10)	22.4 (2.5)	Hot columbiacard task	Number of cards chosen	tDCS (left) ^*^	Anodal lDLPFC, reference rDLPFC, 0.45 mA, 5.3 cm^2^, anodal 0.085 mA/cm^2^, 15 min	1	No effect	Medium
							tDCS (right) ^*^	Anodal rDLPFC, reference lDLPFC, 0.45 mA, 5.3 cm^2^, anodal 0.085 mA/cm^2^, 15 min		Positive effect	
					Cold columbia card task	Number of cards chosen	tDCS (left) ^*^	Anodal lDLPFC, reference rDLPFC, 0.45 mA, 5.3 cm^2^, anodal 0.085 mA/cm^2^, 15 min	1	Positive effect	
							tDCS (right) ^*^	Anodal rDLPFC, reference lDLPFC, 0.45 mA, 5.3 cm^2^, anodal 0.085 mA/cm^2^, 15 min		No effect	
	Gorini et al., 2014	Cocaine (clinical)	18 (10–8)	38.4 (8.2)	Balloon analog risk task	Average numberof pumps onunexplodedballoon	tDCS (left)	Anodal lDLPFC, reference rDLPFC, 1.5 mA, 32 cm^2^, anodal 0.047 mA/cm^2^, 20 min	1	Positive effect	Medium
							tDCS (right)	Anodal rDLPFC, reference lDLPFC, 1.5 mA, 32 cm^2^, anodal 0.047 mA/cm^2^, 20 min	1	Positive effect	
					Game of dice task	Average number of conservative bets	tDCS (left)	Anodal lDLPFC, reference rDLPFC, 1.5 mA, 32 cm^2^, anodal 0.047 mA/cm^2^, 20 min	1	Negative effect	
							tDCS (right)	Anodal rDLPFC, reference lDLPFC, 1.5 mA, 32 cm^2^, anodal 0.047 mA/cm^2^, 20 min		Positive effect	
	Fecteau et al., 2014	Nicotine	12 (5–7)	36.3 (Unknown)	Ultimatum game money	Acceptance rate	tDCS (right)	Anodal rDLPFC, reference lDLPFC, 2 mA, 35 cm^2^, anodal 0.057 mA/cm^2^, 30 min	5	No effect	High
					Ultimatum game cigarettes					Positive effect	
					Risk task money	Choice of low risk vs.high risk options	tDCS (right)	Anodal rDLPFC, reference lDLPFC, 2 mA, 35 cm^2^, anodal 0.057 mA/cm^2^, 30 min	5	No effect	
					Risk task cigarettes					No effect	
	Boggio et al., 2010	Marijuana	25 (15–10)	22.8 (2.6)	Risk task	Percentage lowrisk choice	tDCS (left)^*^	Anodal lDLPFC, reference rDLPFC, 2 mA, 35 cm^2^, anodal 0.057 mA/cm^2^, 15 min	1	Negative effect	High
						Decision time				No effect	
					Risk task	Percentage lowrisk choice	tDCS (right)^*^	Anodal rDLPFC, reference lDLPFC, 2 mA, 35 cm^2^, anodal 0.057 mA/cm^2^, 15 min	1	Negative effect	
						Decision time				No effect	
	Sheffer et al., 2013	Nicotine	16 (unknown)	Unknown	Risk task	Total points earned	HF-rTMS (left)	10 Hz & 20 Hz, 110% RMT,90 trains & 45 trains	1	No effect	Medium
						Total time to completetask				No effect	
Cognitive bias	den Uyl et al., 2015	Alcohol	41 (15–26)	21.7 (3.0)	Affective implicit association task	Reaction time	tDCS (left)	Anodal lDLPFC, reference right supra-orbital region, 1 mA, 35 cm^2^, anodal 0.029 mA/cm^2^, 10 min	1	Positive effect	High
						Bias score (accuracy)				No effect	
					Motivation implicit association task	Reaction time				No effect	
						Bias score (accuracy)				No effect	
	den Uyl et al., 2016a	Alcohol (clinical)	91 (61–30)	47 (8.8)	Alcohol approach task	Approach bias	tDCS (left) (*+ CBM*)	Anodal lDLPFC, reference rDLPFC, 2 mA, 35 cm^2^, anodal 0.057 mA/cm^2^, 20 min	8	No effect	High
	den Uyl et al., 2016b	Alcohol	78 (27–51)	21.8 (3.2)	Alcohol approach task	Approach bias	tDCS (left) (*+ CBM*)	Anodal lDLPFC, reference right supra-orbital region, 1 mA, 35 cm^2^, anodal 0.029 mA/cm^2^, 15 min	3	No effect	High
					Implicit association task	Approach associationbias	tDCS (left) (*+ CBM*)			No effect	
Overall executive functioning	Da Silva et al., 2013	Alcohol (clinical)	13 (13–0)	49^##^	Frontal assessment battery	Total score	tDCS (left)	Anodal lDLPFC, reference right supradeltoid area, 2 mA, 35 cm^2^, anodal 0.057 mA/cm^2^, 20 min	5	Positive effect	High
	Klauss et al., 2014	Alcohol (clinical)	33 (32–1)	44.8 (8.3)	Frontal assessment battery	Total score	tDCS (right)	Anodal rDLPFC, reference lDLPFC, 2 mA, 35 cm^2^, 0.057 mA/cm^2^, 2 × 13 min	5	No effect	High

*Some studies address the effect of non-invasive neuromodulation on several executive functioning domains and are therefore reported multiple times in this table. When one study assessed the effect of left as well as right stimulation this was done in two separate sessions. Results for both protocols were the same when compared to sham and are therefore reported in the same line of the table. lDLPFC, left DLPFC; rDLPFC, right DLPFC; MT, motor threshold; RMT, resting motor threshold*.

# *This number represents the mean of the active treatment group*;

## *median age per group*;

* *online (task performance during stimulation) stimulation protocol*;

∧∧ *with this protocol participants are stimulated with 20 Hz (45 trains) or 10 Hz (90 trains). Colors in the results column indicate a positive effect (green), no effect (blue) or negative effect (red)*.

### Synthesized findings

**Figure 2:**
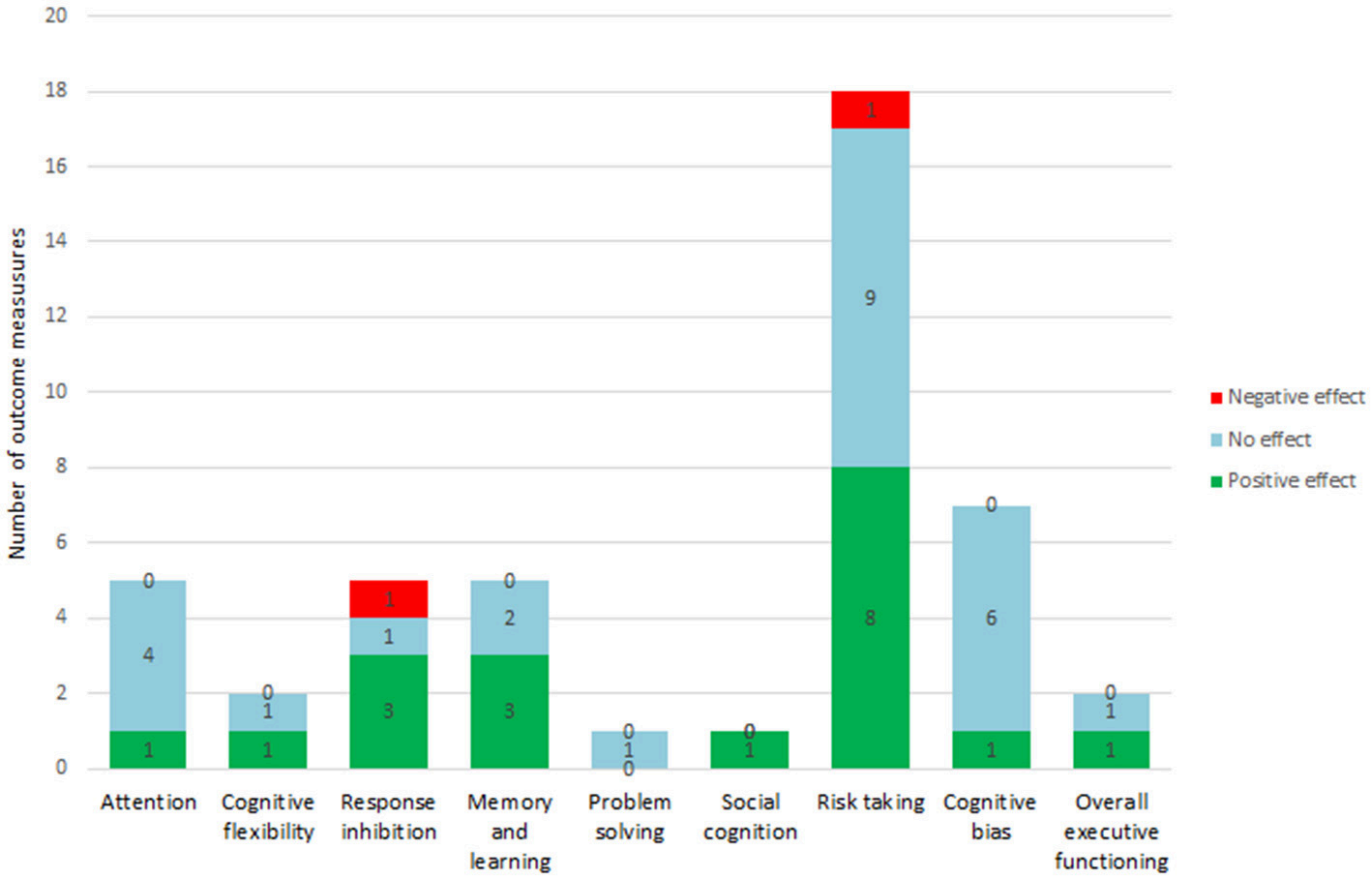
Summary by executive functioning domain. Numbers depicted in the bars indicate the number of outcome measures with a positive, no or negative effect.

The search resulted in the following executive or other cognitive functioning domains: attention (2 papers), cognitive flexibility (2 papers), response inhibition (2 papers), memory and learning (2 papers), problem solving (1 paper), social cognition (1 paper), risk taking (5 papers), cognitive bias modification (3 papers) and overall executive functioning (2 papers). Some studies addressed the effect of multiple stimulation protocols (for example left as well as right anodal tDCS) (3 papers) or of multiple outcome measures [for example multiple tasks (9 papers) or one task with multiple outcome measures (5 papers)] or a combination of these two factors (3 papers). There was a high variability between studies concerning the stimulation protocols applied. As exploration and way of summarizing the literature we calculated the number of different outcome measures and whether they were positively, negatively or not affected. In total thesixteen included studies reported a total of 46 different outcome measures. Overall the effect of non-invasive neuromodulation techniques on these outcome measures were diverse, 17 outcome measures were positively affected, 25 outcome measures were not affected and 4 were negatively affected. A summary of these studies and details about the effects of non-invasive neuromodulation on the outcome measures (i.e., positive, no or negative effect) can be found in Table 1 and Figure 2. The results of these studies are shortly described per domain in the following sections. Studies that assessed performance on multiple tasks that address different executive functioning domains, are discussed in the corresponding sections.

#### Attention

Two studies assessed the effect of non-invasive neuromodulation on attentional processes. One study used the Visual Attention Task, with similarities to a Go-NoGo task (Xu et al., 2013). The other study used a Go-NoGo task adopted from a test battery assessing attentional performance (Herremans et al., 2013). In total these studies assessed five outcome measures. Results of the two studies indicate an effect of HF-rTMS over the right DLPFC on intra-individual reaction time variability (namely a decrease), suggesting improved attention on this Go/NoGo task. The other four outcome measures were not influenced by non-invasive neuromodulation (see Table 1).

#### Cognitive flexibility

The current systematic search revealed two studies assessing the effect of non-invasive neuromodulation on cognitive flexibility. One study used a computerized version of the Wisconsin Card Sorting Task (WCST) (Huang et al., 2016) and the other study used the Numeric Stroop Task (Del Felice et al., 2016), resulting in two outcome measures. HF-rTMS over the left DLPFC did not influence performance on the WCST whereas it increased correct responses during the incongruent trials indicating improved functioning on the Numeric Stroop Task (see Table 1).

#### Response inhibition

The systematic search revealed two studies addressing the effect of non-invasive neuromodulation on response inhibition. One study used a Go-NoGo Task (Del Felice et al., 2016) while the other study used a Delay Discounting Task with money and cigarettes as rewards (Sheffer et al., 2013). In total these studies assessed five outcome measures. HF-rTMS over the left DLPFC had a positive effect on three outcome measures, namely improved accuracy on the Go-NoGo Task and decreased discounting rates for monetary and cigarette gains. Moreover, HF-rTMS increased discounting rates for monetary losses, suggesting a negative effect on one outcome measure. Discounting rates for cigarette losses was not influenced by non-invasive neuromodulation (see Table 1).

#### Memory and learning

Two studies addressed the effect of non-invasive neuromodulation on memory and learning capacity. The first study used the International Shopping List Task, an N-back Task (2-back version) and the Continuous Paired Association Learning Task (Su et al., 2017). The second study used a revised version of the Hopkins Verbal Learning Test and the Brief Visuospatial Memory Test (Qiao et al., 2016). These studies assessed a total of five outcome measures, with a positive effect in three measures and no effect in two measures. Specifically, HF-rTMS over the left DLPFC increased the number of correct responses during the International Shopping List Task indicating improved performance, while it did not change performance on the N-back and Continuous Paired Associated Learning Task. HF-rTMS over the right DLPFC increased the total score during the Hopkins Verbal Learning Test as well as the Brief Visuospatial Memory Test indicating improved performance (see Table 1).

#### Problem solving

One study addressed the effect of non-invasive neuromodulation on problem solving and used the Groton Maze Learning Task with one outcome measure (Su et al., 2017). Multiple sessions of HF-rTMS over the DLPFC did not influence the number of errors during the Groton Maze Learning Task indicating nochange in problem solving capacity (see Table 1).

#### Social cognition

The systematic search revealed one study addressing the effect of non-invasive neuromodulation on social cognition (Su et al., 2017), using the Social Emotional Cognition task with one outcome measure. Results of this study revealed increased proportion of correct responses indicating improved social cognition after multiple sessions of HF-rTMS over the left DLPFC (see Table 1).

#### Risk taking

Five studies addressed the effect of non-invasive neuromodulation on risk taking using several different tasks. The first study used the Hot and Cold version of the Columbia Card Task (Pripfl et al., 2013), while another study used the Balloon Analog Risk Task and the Game of Dice Task (Gorini et al., 2014). Furthermore, one study used the Ultimatum Game and the Risk Task, both with monetary outcomes as well as cigarette outcomes (Fecteau et al., 2014). Finally, two studies used the Risk Task with points as outcome (Boggio et al., 2010; Sheffer et al., 2013). In total 18 outcome measures were assessed. Eight of the outcome measures were positively affected, nine of them were not affected and one was negatively affected. Overall anodal tDCS over the left DLPFC decreased the number of cards chosen on the Cold Columbia Card Task and decreased the number of pumps on the unexploded balloon during the Balloon Analog Risk Task, indicating reduced risky decision making. Furthermore, anodal tDCS over the right DLPFC decreased number of cards chosen during the Hot Columbia Card Task, decreased the number of pumps on the unexploded balloon during the Balloon Analog Risk Task and increased the number of conservative bets during the Game of Dice Task, all indicating reduced risk taking. On the other hand decreased number of conservative bets during the Game of Dice Task was found after one session of tDCS over the left DLPFC, indicating increased risky decision making. Finally, HF-rTMS over the DLPFC did not affect risky decision-making during the Risk Task (see Table 1).

#### Cognitive bias

If non-invasive neuromodulation improves executive functioning one may hypothesize that cognitive bias (i.e., the implicit measure of automatic cognitive motivational processes; Wiers et al., 2007) decreases. The systematic search revealed three studies addressing the effect of non-invasive neuromodulation on cognitive biases. One study used the affective and motivation versions of the Implicit Association Task (den Uyl et al., 2015). Another study used the Alcohol Approach Task (den Uyl et al., 2016a), while the final study used both the Alcohol Approach Task and the Implicit Association Task (den Uyl et al., 2016b). In total seven outcome measures were assessed. No effect of anodal tDCS over the left DLPFC was found on alcohol association bias or alcohol approach bias (six outcome measures), although one study reported a decrease in reaction time during performance of the Affective Implicit Association Task after anodal tDCS over the left DLPFC implicating improved executive functions (den Uyl et al., 2015) (see Table 1).

#### Overall executive functioning

Two studies addressed the effect of non-invasive neuromodulation on overall executive functioning by means of the Frontal Assessment Battery (Da Silva et al., 2013; Klauss et al., 2014). Anodal tDCS over the left DLPFC resulted in a trend significant increase in executive functioning (Da Silva et al., 2013), while anodal stimulation over the right DLPFC did not result in changed executive functioning (Klauss et al., 2014) (see Table 1).

## Discussion

### Summary of main findings

The current systematic review aimed to review and discuss the current evidence of the effects of non-invasive neuromodulation on executive functioning in addictive disorders. Therefore, a systematic search was performed which resulted in 16 studies addressing the following executive and other cognitive functioning domains: attention, cognitive flexibility, response inhibition, memory & learning, problem solving, social cognition, risk taking, cognitive bias and overall executive functioning. As exploration and way of summarizing the literature we calculated the number of different outcome measures within these domains and whether they were positively, negatively or not affected. This resulted in 46 outcome measures that were assessed. Important to note is the large variability of stimulation protocols and study designs used, complicating comparability of the results. For this reason it was impossible to perform a meta-analyses.

The distribution of outcome measures per executive functioning domain was not equal. Risk taking was the best represented, followed by cognitive bias, response inhibition, memory & learning, overall executive functioning and attention. Less represented were cognitive flexibility, problem solving and social cognition. Therefore, considering the proportion of positive effects vs. no and negative effects within an executive function domain as a summarizing method can only be considered explorative. Non-invasive neuromodulation seems to be most effective in social cognition (1 out of 1 outcome measures positive), memory & learning (3 out of 5 outcome measures positive), response inhibition (3 out of 5 outcome measures positive) cognitive flexibility (1 out of 2 outcome measures positive) and overall executive functioning (1 out of 2 outcome measures positive). Non-invasive neuromodulation seems less effective in risk taking (8 out of 18 outcome measures positive), attention (1 out of 5 outcome measures positive) and cognitive bias (1 out of 7 outcome measures positive). No effect of non-invasive neuromodulation was observed on problem solving. This suggests differential effects of non-invasive neuromodulation on different executive and cognitive functions, but more studies are needed as the existing studies are limited in number.

The results of these studies however should be interpreted in light of the goal of neuromodulation, namely improving current treatment. In this light one executive functioning domain may be more relevant compared to other domains. Especially response inhibition may be of high relevance since impulsivity (e.g., diminished response inhibition) is related to poorer treatment outcome (Stevens et al., 2014) and re-initiation of substance use in individuals trying to abstain (de Wit, 2009). Therefore, improving inhibitory functions may prevent an individual from initiating substance use again, and hereby reduce the chance for relapse. Attention and memory & learning may also be relevant domains because abstinence goals and strategies taught during treatment should be remembered (Domínguez-Salas et al., 2016). Cognitive flexibility may be relevant in order to apply the learned strategies and change habitual behavior (Domínguez-Salas et al., 2016). In this light the effect of non-invasive neuromodulation on executive functioning seems to be promising for substance dependence treatment. Unfortunately it is still uncommon to also include outcome measures assessing substance use after non-invasive neuromodulation interventions. From the studies included in the current review seven included at least one measure of substance use and/or relapse (Da Silva et al., 2013; Sheffer et al., 2013; Fecteau et al., 2014; Klauss et al., 2014; Del Felice et al., 2016; den Uyl et al., 2016a; Huang et al., 2016). However, for studies applying only one session it is debatable whether a change in substance use can be expected, and we therefore only discuss the relation of neuromodulation effects on executive functions and clinical outcome measures for studies including multiple neuromodulation sessions. Three of the studies found no effect of non-invasive neuromodulation on executive functions, but did find a decrease in substance use (Klauss et al., 2014; den Uyl et al., 2016a; Huang et al., 2016). Contrary, another study did find a positive effect on executive functioning but no effect on substance use (Del Felice et al., 2016). Furthermore, one study found a trend positive effect on executive functioning, but a trend significant increase in relapse (Da Silva et al., 2013). Based on these studies it is hard to draw any conclusions on the relationship between executive functioning and relapse. Therefore, it is important that future studies also include substance use measures in order to be able to relate effects of neuromodulation on executive functioning to substance use or treatment outcomes.

Besides the different effects of non-invasive neuromodulation on the executive functioning domains there are some other factors which are relevant. First, the type of neuromodulation may influence the extent of the effect. For instance, anodal tDCS only decreases the membrane potential, hereby enhancing neuronal excitability, whereas HF-rTMS actually depolarizes neurons and therefore induces activation of the stimulated tissue (Hummel and Cohen, 2006). Secondly, the side (left vs. right side) of stimulation could differentially contribute to the effect. For example it is postulated that the left DLPFC is related more to approach behavior and impulsive behavior whereas the right DLPFC is related more to avoidance behavior, or control behavior (Miller et al., 2013). This indicates that left sided stimulation may increase impulsive behavior in addicted populations. Indeed, HF-rTMS over the left DLPFC increases responsiveness toward rewarding stimuli (Ahn et al., 2013), suggesting a decrease in inhibitory functioning due to left-sided HF-rTMS. Therefore, stimulation side is important to consider when setting up neuromodulation studies in addictive disorders. Finally, the number of stimulation sessions may contribute to the effect of non-invasive neuromodulation. Applying multiple sessions of non-invasive neuromodulation could induce a summation of effects of single session stimulation (Valero-Cabré et al., 2008), thereby resulting in changes in executive functioning whereas a single session may not have a sufficiently strong effect on long-term improvement of executive functioning.

Altogether, taking the above described contributing factors into account, future studies are needed that compare the effect of different types of stimulation, stimulation side (left vs. right) and number of stimulation sessions on executive functioning and addiction related outcome measures. Additionally, to understand which subgroups within addictive disorders (e.g., high impulsive at baseline) may benefit most from neuromodulation, clinical trials are needed which focus on potential working mechanisms of neuromodulation and patient characteristics (e.g., by including executive functioning tasks and questionnaires at baseline). Such studies may pave the way toward more personalized medicine for addicted subgroups for whom the current treatment does not suffice.

## Limitations

Because of the limited number of studies, which also varied largely in stimulation protocols used, it is not possible to draw firm conclusions on the effectivity of non-invasive neuromodulation on executive functioning in addictive disorders.

## Conclusions

Despite the above described limitation, the results of the studies included in the current review indicate that at least some executive functions may improve through non-invasive neuromodulation, but more research is needed in order to come to a conclusive statement. Before non-invasive neuromodulation can be considered as add-on treatment for substance dependence in the future it first needs to be examined thoroughly in randomized clinical trials, in which both effects on executive functions and effects on addiction outcome measures (i.e., abstinence or craving) are included. Ultimately the development of clinically relevant stimulation protocols may lead to approved clinical application of neuromodulation in treatment of addictive disorders, similar to the approved status of rTMS for treatment of refractory depression for instance (Cusin and Dougherty, 2012). Given the fact that this review shows that non-invasive neuromodulation affects at least some aspects of executive or other cognitive functions in addictive disorders, treatment with non-invasive neuromodulation may ultimately lead to improved clinical treatment outcomes.

## Author contributions

RS, RvH, and AG contributed to the design of the study. JD performed the systematic search. RS selected articles and wrote the first draft of the manuscript. RvH selected articles and revised manuscript and AG revised manuscript. All authors read and approved the final manuscript.

### Conflict of interest statement

The authors declare that the research was conducted in the absence of any commercial or financial relationships that could be construed as a potential conflict of interest.
