# Supplementary material for: Effects of Non-invasive Neuromodulation on Executive and Other Cognitive Functions in Addictive Disorders: A Systematic Review
Source: Front Neurosci. 2018 Sep 19;12:642. doi: 10.3389/fnins.2018.00642 (PMC6156514; doi:10.3389/fnins.2018.00642)
Supplement: Supplementary file 1 [file Table_1.DOCX]

| Study | 1 | 2 | 3 | 4 | 5 | 6 | 7 | 8 | 9 | 10 | 11 | Total | Quality |
| --- | --- | --- | --- | --- | --- | --- | --- | --- | --- | --- | --- | --- | --- |
| Boggio et al., 2010 | 1 | 1 | 1 | 1 | 1 | 1 | 1 | 1 | 1 | 1 | 0 | 10 | high |
| Da Silva et al., 2013 | 1 | 1 | 1 | 1 | 1 | 0 | 0 | 1 | 1 | 1 | 1 | 9 | high |
| Del Felice et al., 2016 | 1 | 1 | 1 | 1 | 1 | 0 | 1 | 1 | 1 | 1 | 0 | 9 | high |
| Den Uyl et al., 2016 (add bio) | 1 | 1 | 1 | 1 | 1 | 1 | 1 | 1 | 1 | 1 | 1 | 11 | high |
| Den Uyl et al., 2016 (alcoholism) | 1 | 1 | 1 | 1 | 1 | 1 | 1 | 1 | 1 | 1 | 1 | 11 | high |
| Den Uyl et al., 2015 | 1 | 1 | 1 | 1 | 1 | 1 | 0 | 1 | 1 | 1 | 1 | 10 | high |
| Fecteau et al., 2014 | 1 | 1 | 1 | 0 | 1 | 1 | 1 | 1 | 1 | 1 | 1 | 10 | high |
| Gorini et al., 2014 | 1 | 1 | 0 | 1 | 1 | 0 | 0 | 1 | 1 | 1 | 0 | 7 | medium |
| Herremans et al., 2013 | 1 | 1 | 0 | 1 | 1 | 0 | 0 | 0 | 1 | 1 | 1 | 7 | medium |
| Huang et al., 2016 | 1 | 1 | 1 | 1 | 1 | 1 | 1 | 1 | 1 | 1 | 1 | 11 | high |
| Klauss et al., 2014 | 1 | 1 | 1 | 1 | 1 | 0 | 1 | 1 | 1 | 1 | 1 | 10 | high |
| Pripfl et al., 2013 | 1 | 0 | 0 | 1 | 1 | 0 | 0 | 1 | 1 | 1 | 1 | 7 | medium |
| Qiao et al., 2016 | 1 | 1 | 1 | 1 | 1 | 1 | 1 | 1 | 1 | 1 | 0 | 10 | high |
| Sheffer et al., 2013 | 1 | 0 | 0 | 0 | 1 | 0 | 0 | 0 | 1 | 1 | 1 | 5 | medium |
| Su et al., 2017 | 1 | 1 | 1 | 1 | 1 | 1 | 1 | 1 | 1 | 1 | 1 | 11 | high |
| Xu et al., 2013 | 1 | 0 | 0 | 1 | 1 | 0 | 0 | 1 | 1 | 1 | 1 | 7 | medium |

**Online supplementary data**

**Table 1:** Quality assessment ratings derived from the PEDro checklist.

Questions:

1 = eligibility criteria were specified

2 = subjects were randomly allocated to groups (in a crossover study, subjects were randomly allocated an order in which treatments were received)

3 = allocation was concealed

4 = the groups were similar at baseline regarding the most important prognostic indicators

5 = there was blinding of all subjects

6 = there was blinding of all therapists who administered the therapy

7 = there was blinding of all assessors who measured at least one key outcome
8 = measures of at least one key outcome were obtained from more than 85% of the subjects initially allocated to groups

9 = all subjects for whom outcome measures were available received the treatment or control condition as allocated or, where this was not the case, data for at least one key outcome was analyzed by "intention to treat"

10 = the results of between statistical comparisons are reported for at least one key outcome

11 = the study provides both point measurements and measurements of variability for at least one key outcome

Quality levels were defined as high (8-11 points), medium (4-7 points) and low (1-3).
