# Supplementary material for: Effects of Non-invasive Neuromodulation on Executive and Other Cognitive Functions in Addictive Disorders: A Systematic Review
Source: Front Neurosci. 2018 Sep 19;12:642. doi: 10.3389/fnins.2018.00642 (PMC6156514; doi:10.3389/fnins.2018.00642)
Supplement: Supplementary file 3 [file Data_Sheet_2.doc]

**Online supplement 2**

**Detailed search strategy**

All searches were run 18 October 2017.

*Ovid MEDLINE(R) Epub Ahead of Print, In-Process & Other Non-Indexed Citations, Ovid MEDLINE(R) Daily and Ovid MEDLINE(R) <1946 to Present>*

| 1 | gambling/ |
| --- | --- |
| 2 | (gambling or gambler?).ab,kf,ti |
| 3 | exp substance related disorders/ or alcoholism/ or alcohol drinking/ or eating disorders/ or binge-eating disorder/ or bulimia/ or (smoking/ and (abuse or misuse or depend* or addict*)) |
| 4 | (((substance or polydrug? or drug? or alcohol or nicotin? or marihuana or marijuana or cannabis or pot or hashish or heroin or speed or cocain or crack or mdma or ketamine or amphetamine or methamphetamine or crystal meth or tobacco or smoking) adj3 (abuse or misuse or depend* or addict*)) or Hydroxybutyric acid or gamma-hydroxybutyrate or gammahydroxybutyrate or γ-Hydroxybutyrate or GHB or hydroxybutanoic acid or y butyrolactone or gamma butyrolactone or gammabutyrolactone or gbl or binge drink* or problem drink* or binge eat* or bulimia).ab,jw,kf,ti |
| 5 | (abuse or misuse or addiction or dependence).jw |
| 6 | or/1-5 [P - substance abuse] |
| 7 | prefrontal cortex/ |
| 8 | (prefrontal or pre frontal or Medial Frontal Gyrus or Orbitofrontal or Superior Frontal Gyrus or Inferior Frontal Gyrus or orbital cortex).ab,kf,ti |
| 9 | or/7-8 [prefrontal cortex] |
| 10 | transcranial direct current stimulation/ or transcranial magnetic stimulation/ |
| 11 | ((transcranial adj3 stimulation) or tms or rtms or tdcs).ab,kf,ti. |
| 12 | electrostimulation.mp |
| 13 | or/10-12 [noninvasive neuromodulation] |
| 14 | and/6,9,13 [I prefrontal cortex neurostimulation] |
| 15 | executive function/ or stroop test/ or trail making test/ |
| 16 | (executive function* or executive control or cognitive control).ab,kf,ti |
| 17 | (flexibility and cogn*).ab,kf,ti |
| 18 | (switch* adj10 (task? or rule?)).ab,kf,ti |
| 19 | ((wisconsin adj5 (test or taks)) or set shift or (Inhibit* adj3 (response or control or task?)) or interference inhibition or ((nogo or go) adj5 (task* or test* or signal*)) or Simon or Stroop or Stop signal or Delay discounting or trail making or "tower of london").ab,kf,ti |
| 20 | or/15-19 [executive functioning] |
| 21 | memory, short-term/ or verbal learning/ |
| 22 | (Working memory or shortterm memory or short term memory or verbal learning or visuospatial memory or Complex calculation or PASAT or paced auditory serial addition task or "Delayed match to sample" or "N-back" or AX-CPT or continuous performance task or Spatial span or sequence recall or Sternberg task or Spatial working memory task or word generation).ab,kf,ti |
| 23 | or/21-22 [executive functioning | working memory] |
| 24 | 9 and 13 and (20 or 23) |
| 25 | 14 or 24 |
| 26 | Remove duplicates from 25 |

*Ovid* Embase Classic+Embase <1947 to 2017 October 17>

| 1 | *gambling/ |
| --- | --- |
| 2 | (gambling or gambler?).ab,kw,ti |
| 3 | exp drug dependence/ or eating disorder/ or binge-eating disorder/ or bulimia/ or food addiction/ or (smoking/ and (abuse or misuse or depend* or addict*)) |
| 4 | (((substance or polydrug? or drug? or alcohol or nicotin? or marihuana or marijuana or cannabis or pot or hashish or heroin or speed or cocain or crack or mdma or ketamine or amphetamine or methamphetamine or crystal meth or tobacco or smoking) adj3 (abuse or misuse or depend* or addict*)) or Hydroxybutyric acid or gamma-hydroxybutyrate or gammahydroxybutyrate or γ-Hydroxybutyrate or GHB or hydroxybutanoic acid or y butyrolactone or gamma butyrolactone or gammabutyrolactone or gbl or binge drink* or problem drink* or binge eat* or bulimia).ab,jx,kw,ti |
| 5 | (abuse or misuse or addiction or dependence).jx |
| 6 | or/1-5 [P - substance abuse] |
| 7 | *prefrontal cortex/ |
| 8 | (prefrontal or pre frontal or Medial Frontal Gyrus or Orbitofrontal or Superior Frontal Gyrus or Inferior Frontal Gyrus or orbital cortex).ab,kw,ti |
| 9 | or/7-8 [prefrontal cortex] |
| 10 | *transcranial direct current stimulation/ or exp *transcranial magnetic stimulation/ or *electrostimulation/ |
| 11 | ((transcranial adj3 stimulation) or tms or rtms or tdcs).ab,kw,ti. |
| 12 | electrostimulation.mp |
| 13 | or/10-12 [noninvasive neuromodulation] |
| 14 | and/6,9,13 [I prefrontal cortex neurostimulation] |
| 15 | exp *executive function/ |
| 16 | (executive function* or executive control or cognitive control).ab,kw,ti |
| 17 | (flexibility and cogn*).ab,kw,ti |
| 18 | (switch* adj10 (task? or rule?)).ab,kw,ti |
| 19 | ((wisconsin adj5 (test or taks)) or set shift or (Inhibit* adj3 (response or control or task?)) or interference inhibition or ((nogo or go) adj5 (task* or test* or signal*)) or Simon or Stroop or Stop signal or Delay discounting or trail making or "tower of london").ab,kw,ti |
| 20 | or/15-19 [executive functioning] |
| 21 | *working memory/ |
| 22 | (Working memory or shortterm memory or short term memory or verbal learning or visuospatial memory or Complex calculation or PASAT or paced auditory serial addition task or "Delayed match to sample" or "N-back" or AX-CPT or continuous performance task or Spatial span or sequence recall or Sternberg task or Spatial working memory task or word generation).ab,kw,ti |
| 23 | or/21-22 [executive functioning | working memory] |
| 24 | 9 and 13 and (20 or 23) |
| 25 | 14 or 24 |
| 26 | Remove duplicates from 25 |

*Ovid* PsycINFO <1806 to October Week 2 2017>

| 1 | exp gambling/ |
| --- | --- |
| 2 | (gambling or gambler?).ab,id,ti |
| 3 | drug abuse/ or exp alcohol abuse/ or exp drug dependency/ or bulimia/ or binge eating disorder/ or (tobacco smoking/ and (abuse or misuse or depend* or addict*)) |
| 4 | (((substance or polydrug? or drug? or alcohol or nicotin? or marihuana or marijuana or cannabis or pot or hashish or heroin or speed or cocain or crack or mdma or ketamine or amphetamine or methamphetamine or crystal meth or tobacco or smoking) adj3 (abuse or misuse or depend* or addict*)) or Hydroxybutyric acid or gamma-hydroxybutyrate or gammahydroxybutyrate or γ-Hydroxybutyrate or GHB or hydroxybutanoic acid or y butyrolactone or gamma butyrolactone or gammabutyrolactone or gbl or binge drink* or problem drink* or binge eat* or bulimia).ab,jx,id,ti |
| 5 | (abuse or misuse or addiction or dependence).jx |
| 6 | or/1-5 [P - substance abuse] |
| 7 | prefrontal cortex/ |
| 8 | (prefrontal or pre frontal or Medial Frontal Gyrus or Orbitofrontal or Superior Frontal Gyrus or Inferior Frontal Gyrus or orbital cortex).ab,id,ti |
| 9 | or/7-8 [prefrontal cortex] |
| 10 | electrical stimulation/ or transcranial magnetic stimulation/ |
| 11 | ((transcranial adj3 stimulation) or tms or rtms or tdcs).ab,id,ti. |
| 12 | electrostimulation.mp |
| 13 | or/10-12 [noninvasive neuromodulation] |
| 14 | and/6,9,13 [I prefrontal cortex neurostimulation] |
| 15 | exp executive function/ |
| 16 | (executive function* or executive control or cognitive control).ab,id,ti |
| 17 | (flexibility and cogn*).ab,id,ti |
| 18 | (switch* adj10 (task? or rule?)).ab,id,ti |
| 19 | ((wisconsin adj5 (test or taks)) or set shift or (Inhibit* adj3 (response or control or task?)) or interference inhibition or ((nogo or go) adj5 (task* or test* or signal*)) or Simon or Stroop or Stop signal or Delay discounting or trail making or "tower of london").ab,id,ti,tm |
| 20 | or/15-19 [executive functioning] |
| 21 | short term memory/ |
| 22 | (Working memory or shortterm memory or short term memory or verbal learning or visuospatial memory or Complex calculation or PASAT or paced auditory serial addition task or "Delayed match to sample" or "N-back" or AX-CPT or continuous performance task or Spatial span or sequence recall or Sternberg task or Spatial working memory task or word generation).ab,id,ti,tm |
| 23 | or/21-22 [executive functioning | working memory] |
| 24 | 9 and 13 and (20 or 23) |
| 25 | 14 or 24 |
| 26 | Remove duplicates from 25 |
